# Crystal structure of [*N*,*N*′-bis­(4-methyl­phen­yl)-1,2-di­phenyl­ethane-1,2-di­imine-κ^2^ *N*,*N*′]di­chlorido­palladium(II) methanol monosolvate

**DOI:** 10.1107/S2056989015014851

**Published:** 2015-08-15

**Authors:** Alfredo Peñaloza, Frank R. Fronczek, Ralph Isovitsch

**Affiliations:** a 13406 Philadelphia Street, Whittier College, Department of Chemistry, Whittier College, Whittier, CA 90601, USA; b Department of Chemistry, Louisiana State University, Baton Rouge, LA 70803, USA

**Keywords:** crystal structure, palladium(II) di­chlorido di­imine complex, polymerization catalyst

## Abstract

The title compound, [PdCl_2_(C_28_H_24_N_2_)]·CH_3_OH, was pre­pared from the reaction of PdCl_2_(DMSO)_2_ (DMSO is di­methyl sulfoxide) and *N*,*N′*-bis­(4-methyl­phen­yl)-1,2-di­phenyl­ethane-1,2-di­imine in methanol. The chelating di­imine core of the title compound deviates slightly from planarity, with an N—C—C—N torsion angle of 5.3 (3)°. Delocalization in the di­imine core is indicated by N—C and C—C bonds that are, respectively, longer and shorter than those found in related nonchelating di­imines. The distorted square-planar coordination environment around the Pd^II^ atom is manifested as bond angles that are smaller and larger than 90°, and palladacycle torsion angles of −173.22 (16) and 167.06 (16)°. These deviations are attributed to the small bite angle of 79.13 (8)° of the di­imine chelate. The crystal packing exhibits weak inter­molecular hydrogen-bonding inter­actions involving aromatic H atoms, Cl atoms and inter­calated methanol solvent mol­ecules, defining layers parallel to (010).

## Related literature   

Palladium(II) diimiine complexes have been widely used as polymerization catalysts for α-olefins (Johnson *et al.*, 1995 ▸; Popeney & Guan, 2005 ▸) and are prepared easily by the reaction of PdCl_2_(DMSO)_2_ with the di­imine of choice (Kubota *et al.*, 2013 ▸; Ettedgui & Neumann, 2009 ▸; Price *et al.*, 1972 ▸). For structural information about related palladium(II) di­imine complexes, see: Kubota *et al.* (2013 ▸); Comerlato *et al.* (2001 ▸); Dyakonenko *et al.* (2015 ▸). For structures of other di­imines, see: Wang *et al.* (2012 ▸); Zhao *et al.* (2015 ▸).
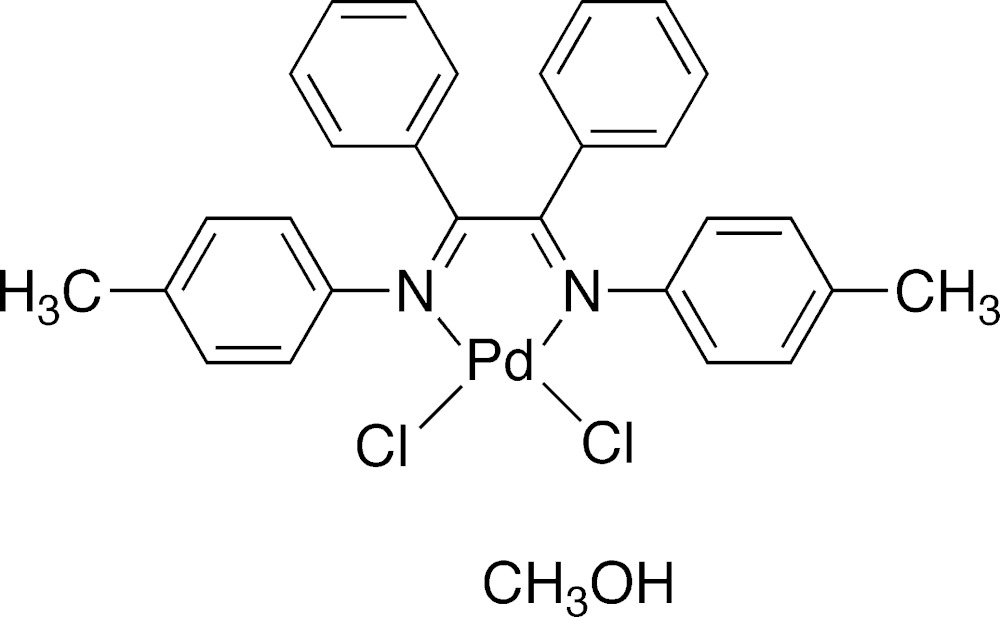


## Experimental   

### Crystal data  

[PdCl_2_(C_28_H_24_N_2_)]·CH_4_O
*M* *_r_* = 597.83
Triclinic, 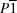

*a* = 8.8213 (3) Å
*b* = 12.3364 (3) Å
*c* = 12.7697 (4) Å
α = 108.992 (2)°
β = 93.900 (3)°
γ = 92.457 (3)°
*V* = 1307.83 (7) Å^3^
*Z* = 2
Mo *K*α radiation
μ = 0.94 mm^−1^
*T* = 90 K
0.18 × 0.10 × 0.06 mm

### Data collection  

Bruker Kappa APEXII DUO CCD diffractometer
Absorption correction: multi-scan (*SADABS*; Bruker, 2014 ▸) *T*_min_ = 0.831, *T*_max_ = 0.946
12258 measured reflections
5965 independent reflections
5026 reflections with *I* > 2σ(*I*)
*R*_int_ = 0.032

### Refinement  

*R*[*F* ^2^ > 2σ(*F* ^2^)] = 0.031
*wR*(*F* ^2^) = 0.066
*S* = 1.02
5965 reflections
322 parameters
1 restraint
H atoms treated by a mixture of independent and constrained refinement
Δρ_max_ = 0.57 e Å^−3^
Δρ_min_ = −0.64 e Å^−3^

### 

Data collection: *APEX2* (Bruker, 2014 ▸); cell refinement: *SAINT* (Bruker, 2014 ▸); data reduction: *SAINT*; program(s) used to solve structure: *SHELXS97* (Sheldrick, 2008 ▸); program(s) used to refine structure: *SHELXL97* (Sheldrick, 2008 ▸); molecular graphics: *ORTEP-3 for Windows* (Farrugia, 2012 ▸); software used to prepare material for publication: *SHELXL97*.

## Supplementary Material

Crystal structure: contains datablock(s) I, New_Global_Publ_Block. DOI: 10.1107/S2056989015014851/wm5193sup1.cif

Structure factors: contains datablock(s) I. DOI: 10.1107/S2056989015014851/wm5193Isup2.hkl

Supporting information file. DOI: 10.1107/S2056989015014851/wm5193sup3.pdf

**Fig. 1.**
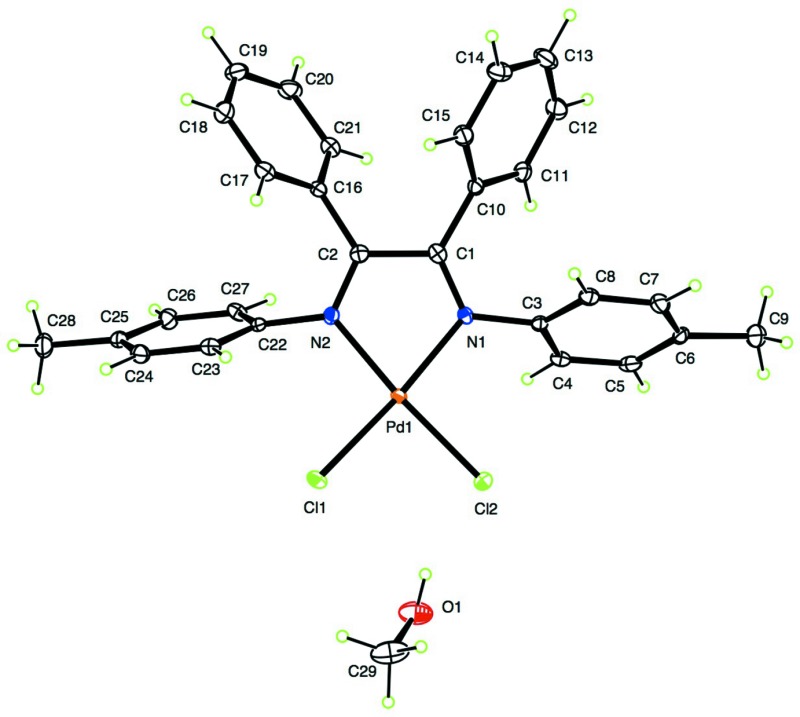
The molecular components of the title compound. Displacement ellipsoids are represented at the 50% probability level.

. DOI: 10.1107/S2056989015014851/wm5193fig1.tif
The mol­ecular components of the title compound. Displacement ellipsoids are represented at the 50% probability level.

**Fig. 2.**
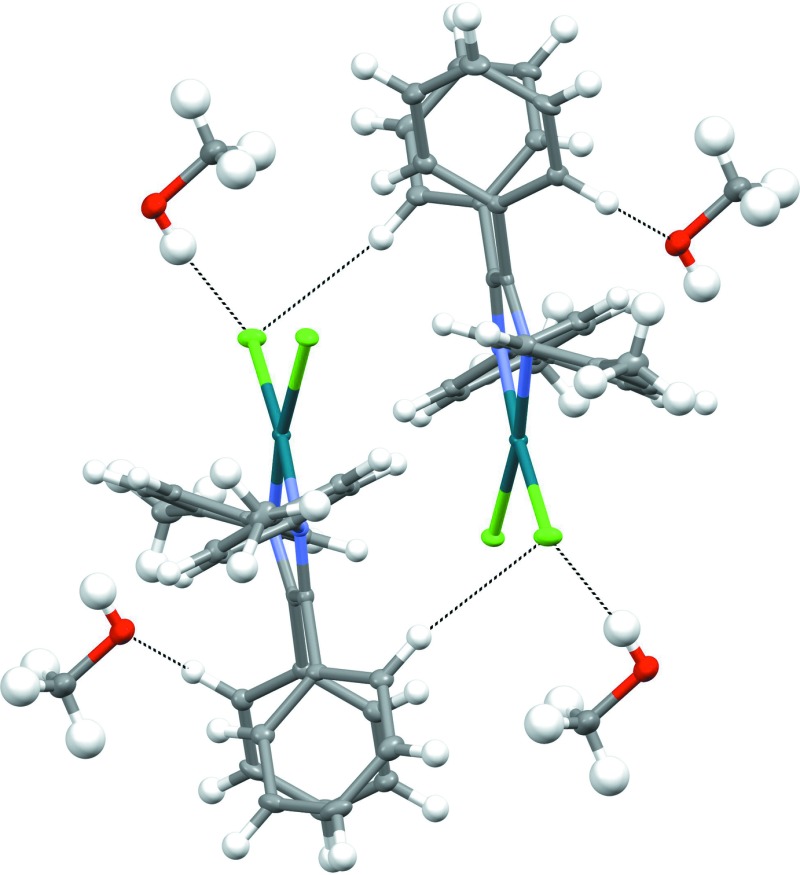
Crystal packing in the title compound, with intermolecular hydrogen bonding emphasized as dashed lines.

. DOI: 10.1107/S2056989015014851/wm5193fig2.tif
Crystal packing in the title compound, with inter­molecular hydrogen bonding emphasized as dashed lines.

**Fig. 3.**
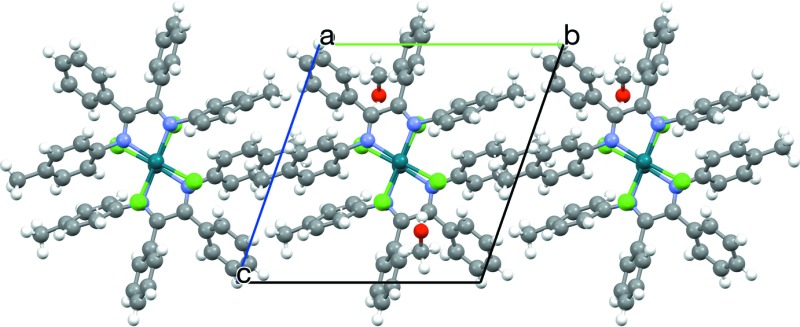
Crystal packing in the title compound as viewed along [100].

. DOI: 10.1107/S2056989015014851/wm5193fig3.tif
Crystal packing in the title compound as viewed along [100].

CCDC reference: 1417572

Additional supporting information:  crystallographic information; 3D view; checkCIF report

## Figures and Tables

**Table 1:** Hydrogen-bond geometry (, )

*D*H*A*	*D*H	H*A*	*D* *A*	*D*H*A*
O1H10*H*Cl2	0.83(2)	2.36(2)	3.161(2)	163(3)
C17H17Cl2^i^	0.95	2.80	3.708(3)	161
C21H21O1^ii^	0.95	2.48	3.275(3)	141

Symmetry codes: (i) 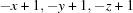; (ii) 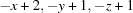.

## supplementary crystallographic information

### S1. Synthesis and crystallization

0.086 g (0.257 mmol, 1 eq.) of PdCl_2_(DMSO)_2_ and 0.100 g (0.257 mmol, 1 eq.) of *N*,*N'*-di(4-methyl­phenyl)-1,2-di­phenyl­ethane-1,2-di­imine were combined with 10 ml of methanol and stirred for 1.5 hours at room temperature. The orange precipitate that formed was collected *via* vacuum filtration, washed well with water and then air-dried giving 0.0363 g (25%) of the title compound. Slow evaporation of the reaction mixture gave X-ray quality crystals of the title compound. MP: > 532 K. IR (KCl): 3135, 2922, 1514 cm^-1^. UV-Vis (λ nm (ε)): 242 (41,200), 264 (34,800), 317 (17,800). TLC (alumina, ethanol): R*_f_* = 0.59.

### S2. Refinement

H atoms were placed in idealized positions, guided by difference maps, with C—H bond lengths in the range 0.95-0.98 Å, with *U*_iso_(H) = 1.2*U*_eq_(C) of the attached atom (1.5 for methyl), and thereafter treated as riding. A torsional parameter was refined for each methyl group. The H atom of the methanol solvent molecule was refined with O—H = 0.85 Å and *U*_iso_(H) = 1.5*U*_eq_(O).

### Crystal data

**Table:**

[PdCl_2_(C_28_H_24_N_2_)]·CH_4_O	*Z* = 2
*M**_r_* = 597.83	*F*(000) = 608
Triclinic, *P*1	*D*_x_ = 1.518 Mg m^−^^3^
Hall symbol: -P 1	Mo *K*α radiation, λ = 0.71073 Å
*a* = 8.8213 (3) Å	Cell parameters from 4900 reflections
*b* = 12.3364 (3) Å	θ = 2.8–27.5°
*c* = 12.7697 (4) Å	µ = 0.94 mm^−^^1^
α = 108.992 (2)°	*T* = 90 K
β = 93.900 (3)°	Plate, orange
γ = 92.457 (3)°	0.18 × 0.10 × 0.06 mm
*V* = 1307.83 (7) Å^3^	

### Data collection

**Table:**

Bruker Kappa APEXII DUO CCD diffractometer	5965 independent reflections
Radiation source: fine-focus sealed tube	5026 reflections with *I* > 2σ(*I*)
TRIUMPH curved graphite monochromator	*R*_int_ = 0.032
φ and ω scans	θ_max_ = 27.5°, θ_min_ = 1.7°
Absorption correction: multi-scan (*SADABS*; Bruker, 2014)	*h* = −11→11
*T*_min_ = 0.831, *T*_max_ = 0.946	*k* = −16→16
12258 measured reflections	*l* = −16→16

### Refinement

**Table:**

Refinement on *F*^2^	Primary atom site location: structure-invariant direct methods
Least-squares matrix: full	Secondary atom site location: difference Fourier map
*R*[*F*^2^ > 2σ(*F*^2^)] = 0.031	Hydrogen site location: inferred from neighbouring sites
*wR*(*F*^2^) = 0.066	H atoms treated by a mixture of independent and constrained refinement
*S* = 1.02	*w* = 1/[σ^2^(*F*_o_^2^) + (0.0243*P*)^2^ + 0.2447*P*] where *P* = (*F*_o_^2^ + 2*F*_c_^2^)/3
5965 reflections	(Δ/σ)_max_ = 0.002
322 parameters	Δρ_max_ = 0.57 e Å^−^^3^
1 restraint	Δρ_min_ = −0.64 e Å^−^^3^

### Special details

**Table:**

Geometry. All e.s.d.'s (except the e.s.d. in the dihedral angle between two l.s. planes) are estimated using the full covariance matrix. The cell e.s.d.'s are taken into account individually in the estimation of e.s.d.'s in distances, angles and torsion angles; correlations between e.s.d.'s in cell parameters are only used when they are defined by crystal symmetry. An approximate (isotropic) treatment of cell e.s.d.'s is used for estimating e.s.d.'s involving l.s. planes.
Refinement. Refinement of *F*^2^ against ALL reflections. The weighted *R*-factor *wR* and goodness of fit *S* are based on *F*^2^, conventional *R*-factors *R* are based on *F*, with *F* set to zero for negative *F*^2^. The threshold expression of *F*^2^ > σ(*F*^2^) is used only for calculating *R*-factors(gt) *etc*. and is not relevant to the choice of reflections for refinement. *R*-factors based on *F*^2^ are statistically about twice as large as those based on *F*, and *R*- factors based on ALL data will be even larger.

### Fractional atomic coordinates and isotropic or equivalent isotropic displacement parameters (Å^2^)

**Table:**

	*x*	*y*	*z*	*U*_iso_*/*U*_eq_	
Pd1	0.72678 (2)	0.502582 (15)	0.508729 (15)	0.00896 (6)	
Cl1	0.66581 (7)	0.51705 (5)	0.33726 (5)	0.01389 (13)	
Cl2	0.78558 (7)	0.31784 (5)	0.42450 (5)	0.01609 (14)	
N1	0.7550 (2)	0.49790 (16)	0.66557 (16)	0.0092 (4)	
N2	0.6911 (2)	0.66555 (16)	0.59660 (16)	0.0102 (4)	
C1	0.7157 (3)	0.5882 (2)	0.7421 (2)	0.0109 (5)	
C2	0.6855 (3)	0.6871 (2)	0.7029 (2)	0.0114 (5)	
C3	0.8034 (3)	0.39982 (19)	0.69323 (19)	0.0110 (5)	
C4	0.9500 (3)	0.3682 (2)	0.67176 (19)	0.0125 (5)	
H4	1.0122	0.4074	0.6357	0.015*	
C5	1.0047 (3)	0.2789 (2)	0.7035 (2)	0.0151 (5)	
H5	1.1058	0.2580	0.6900	0.018*	
C6	0.9146 (3)	0.2192 (2)	0.7545 (2)	0.0150 (5)	
C7	0.7650 (3)	0.2482 (2)	0.7692 (2)	0.0163 (6)	
H7	0.7002	0.2054	0.8002	0.020*	
C8	0.7082 (3)	0.3385 (2)	0.7394 (2)	0.0130 (5)	
H8	0.6060	0.3578	0.7505	0.016*	
C9	0.9798 (3)	0.1291 (2)	0.7979 (2)	0.0226 (6)	
H9A	1.0674	0.0985	0.7573	0.034*	
H9B	0.9018	0.0667	0.7874	0.034*	
H9C	1.0126	0.1639	0.8773	0.034*	
C10	0.7048 (3)	0.59987 (19)	0.8604 (2)	0.0112 (5)	
C11	0.8257 (3)	0.5791 (2)	0.9252 (2)	0.0142 (5)	
H11	0.9185	0.5560	0.8937	0.017*	
C12	0.8109 (3)	0.5920 (2)	1.0360 (2)	0.0186 (6)	
H12	0.8943	0.5789	1.0805	0.022*	
C13	0.6756 (3)	0.6238 (2)	1.0820 (2)	0.0212 (6)	
H13	0.6658	0.6318	1.1577	0.025*	
C14	0.5547 (3)	0.6441 (2)	1.0180 (2)	0.0184 (6)	
H14	0.4616	0.6654	1.0496	0.022*	
C15	0.5689 (3)	0.6334 (2)	0.9075 (2)	0.0155 (5)	
H15	0.4862	0.6488	0.8641	0.019*	
C16	0.6638 (3)	0.8028 (2)	0.78122 (19)	0.0117 (5)	
C17	0.5369 (3)	0.8595 (2)	0.7631 (2)	0.0158 (5)	
H17	0.4630	0.8228	0.7029	0.019*	
C18	0.5185 (3)	0.9697 (2)	0.8332 (2)	0.0192 (6)	
H18	0.4316	1.0084	0.8210	0.023*	
C19	0.6264 (3)	1.0236 (2)	0.9210 (2)	0.0211 (6)	
H19	0.6145	1.0997	0.9681	0.025*	
C20	0.7514 (3)	0.9665 (2)	0.9400 (2)	0.0192 (6)	
H20	0.8244	1.0031	1.0010	0.023*	
C21	0.7709 (3)	0.8564 (2)	0.8709 (2)	0.0155 (5)	
H21	0.8568	0.8175	0.8844	0.019*	
C22	0.6995 (3)	0.75880 (19)	0.55233 (19)	0.0107 (5)	
C23	0.5828 (3)	0.7719 (2)	0.4801 (2)	0.0130 (5)	
H23	0.4960	0.7188	0.4581	0.016*	
C24	0.5944 (3)	0.8638 (2)	0.4402 (2)	0.0141 (5)	
H24	0.5146	0.8734	0.3908	0.017*	
C25	0.7211 (3)	0.9422 (2)	0.4715 (2)	0.0136 (5)	
C26	0.8371 (3)	0.9256 (2)	0.5424 (2)	0.0149 (5)	
H26	0.9243	0.9783	0.5640	0.018*	
C27	0.8288 (3)	0.8339 (2)	0.5825 (2)	0.0135 (5)	
H27	0.9101	0.8227	0.6298	0.016*	
C28	0.7325 (3)	1.0438 (2)	0.4304 (2)	0.0200 (6)	
H28A	0.8004	1.0280	0.3704	0.030*	
H28B	0.7734	1.1122	0.4917	0.030*	
H28C	0.6311	1.0572	0.4026	0.030*	
O1	0.9588 (2)	0.31675 (18)	0.21549 (16)	0.0275 (5)	
H10H	0.916 (4)	0.333 (3)	0.274 (2)	0.041*	
C29	0.8406 (3)	0.2883 (3)	0.1302 (2)	0.0322 (8)	
H29A	0.7635	0.2362	0.1441	0.048*	
H29B	0.8815	0.2505	0.0589	0.048*	
H29C	0.7942	0.3584	0.1281	0.048*	

### Atomic displacement parameters (Å^2^)

**Table:**

	*U*^11^	*U*^22^	*U*^33^	*U*^12^	*U*^13^	*U*^23^
Pd1	0.01082 (10)	0.00890 (9)	0.00697 (10)	−0.00045 (7)	0.00080 (7)	0.00251 (7)
Cl1	0.0166 (3)	0.0163 (3)	0.0086 (3)	0.0014 (2)	−0.0004 (2)	0.0042 (2)
Cl2	0.0252 (4)	0.0101 (3)	0.0123 (3)	0.0019 (2)	0.0020 (3)	0.0027 (2)
N1	0.0083 (10)	0.0103 (10)	0.0096 (10)	−0.0003 (8)	0.0001 (8)	0.0045 (8)
N2	0.0105 (10)	0.0094 (10)	0.0110 (10)	0.0014 (8)	0.0002 (8)	0.0037 (8)
C1	0.0070 (12)	0.0140 (12)	0.0120 (12)	−0.0020 (9)	−0.0010 (9)	0.0055 (10)
C2	0.0091 (12)	0.0126 (12)	0.0123 (12)	0.0010 (9)	0.0012 (9)	0.0037 (10)
C3	0.0153 (13)	0.0085 (11)	0.0076 (12)	0.0006 (9)	−0.0016 (9)	0.0010 (10)
C4	0.0135 (13)	0.0141 (12)	0.0084 (12)	0.0003 (10)	0.0008 (10)	0.0018 (10)
C5	0.0147 (13)	0.0171 (13)	0.0103 (13)	0.0046 (10)	0.0010 (10)	−0.0001 (10)
C6	0.0247 (15)	0.0090 (12)	0.0095 (12)	0.0046 (10)	−0.0028 (10)	0.0012 (10)
C7	0.0227 (15)	0.0134 (13)	0.0132 (13)	−0.0013 (11)	0.0033 (11)	0.0049 (11)
C8	0.0138 (13)	0.0131 (12)	0.0106 (12)	−0.0003 (10)	0.0001 (10)	0.0021 (10)
C9	0.0314 (16)	0.0203 (14)	0.0182 (15)	0.0105 (12)	0.0013 (12)	0.0083 (12)
C10	0.0138 (13)	0.0089 (12)	0.0107 (12)	−0.0005 (9)	0.0001 (10)	0.0034 (10)
C11	0.0149 (13)	0.0133 (12)	0.0146 (13)	0.0036 (10)	0.0001 (10)	0.0051 (10)
C12	0.0244 (15)	0.0172 (14)	0.0121 (13)	−0.0012 (11)	−0.0066 (11)	0.0038 (11)
C13	0.0306 (16)	0.0221 (14)	0.0113 (13)	−0.0031 (12)	0.0034 (12)	0.0066 (11)
C14	0.0190 (14)	0.0204 (14)	0.0150 (14)	−0.0001 (11)	0.0052 (11)	0.0041 (11)
C15	0.0183 (14)	0.0142 (13)	0.0142 (13)	0.0002 (10)	−0.0001 (10)	0.0053 (11)
C16	0.0159 (13)	0.0110 (12)	0.0087 (12)	0.0009 (10)	0.0025 (10)	0.0036 (10)
C17	0.0188 (14)	0.0179 (13)	0.0114 (13)	0.0026 (10)	0.0016 (10)	0.0057 (11)
C18	0.0222 (15)	0.0187 (14)	0.0194 (14)	0.0086 (11)	0.0055 (11)	0.0084 (12)
C19	0.0324 (17)	0.0136 (13)	0.0165 (14)	0.0031 (12)	0.0103 (12)	0.0023 (11)
C20	0.0252 (15)	0.0165 (13)	0.0124 (13)	−0.0042 (11)	0.0004 (11)	0.0010 (11)
C21	0.0182 (14)	0.0166 (13)	0.0127 (13)	0.0018 (10)	0.0011 (10)	0.0062 (11)
C22	0.0162 (13)	0.0079 (11)	0.0085 (12)	0.0031 (9)	0.0045 (10)	0.0023 (9)
C23	0.0132 (13)	0.0139 (12)	0.0105 (12)	0.0011 (10)	0.0014 (10)	0.0021 (10)
C24	0.0138 (13)	0.0173 (13)	0.0119 (13)	0.0056 (10)	0.0008 (10)	0.0052 (11)
C25	0.0179 (13)	0.0119 (12)	0.0125 (13)	0.0044 (10)	0.0063 (10)	0.0046 (10)
C26	0.0148 (13)	0.0138 (13)	0.0170 (14)	−0.0003 (10)	0.0031 (10)	0.0063 (11)
C27	0.0138 (13)	0.0178 (13)	0.0093 (12)	0.0002 (10)	−0.0004 (10)	0.0053 (10)
C28	0.0232 (15)	0.0182 (14)	0.0243 (15)	0.0064 (11)	0.0077 (12)	0.0129 (12)
O1	0.0199 (11)	0.0366 (12)	0.0214 (11)	0.0001 (9)	0.0066 (9)	0.0027 (10)
C29	0.0231 (17)	0.0367 (18)	0.0260 (17)	−0.0004 (13)	0.0067 (13)	−0.0050 (14)

### Geometric parameters (Å, º)

**Table:**

Pd1—N2	2.0086 (19)	C14—C15	1.388 (3)
Pd1—N1	2.0211 (19)	C14—H14	0.9500
Pd1—Cl2	2.2807 (6)	C15—H15	0.9500
Pd1—Cl1	2.2842 (6)	C16—C17	1.390 (4)
N1—C1	1.299 (3)	C16—C21	1.396 (3)
N1—C3	1.440 (3)	C17—C18	1.386 (4)
N2—C2	1.300 (3)	C17—H17	0.9500
N2—C22	1.439 (3)	C18—C19	1.386 (4)
C1—C10	1.480 (3)	C18—H18	0.9500
C1—C2	1.489 (3)	C19—C20	1.383 (4)
C2—C16	1.481 (3)	C19—H19	0.9500
C3—C4	1.386 (3)	C20—C21	1.383 (4)
C3—C8	1.390 (3)	C20—H20	0.9500
C4—C5	1.386 (3)	C21—H21	0.9500
C4—H4	0.9500	C22—C23	1.386 (3)
C5—C6	1.389 (3)	C22—C27	1.387 (3)
C5—H5	0.9500	C23—C24	1.388 (3)
C6—C7	1.392 (4)	C23—H23	0.9500
C6—C9	1.512 (3)	C24—C25	1.392 (3)
C7—C8	1.390 (3)	C24—H24	0.9500
C7—H7	0.9500	C25—C26	1.386 (4)
C8—H8	0.9500	C25—C28	1.511 (3)
C9—H9A	0.9800	C26—C27	1.387 (3)
C9—H9B	0.9800	C26—H26	0.9500
C9—H9C	0.9800	C27—H27	0.9500
C10—C11	1.389 (3)	C28—H28A	0.9800
C10—C15	1.396 (3)	C28—H28B	0.9800
C11—C12	1.387 (3)	C28—H28C	0.9800
C11—H11	0.9500	O1—C29	1.400 (4)
C12—C13	1.382 (4)	O1—H10H	0.831 (17)
C12—H12	0.9500	C29—H29A	0.9800
C13—C14	1.380 (4)	C29—H29B	0.9800
C13—H13	0.9500	C29—H29C	0.9800
			
N2—Pd1—N1	79.13 (8)	C13—C14—H14	119.9
N2—Pd1—Cl2	173.82 (6)	C15—C14—H14	119.9
N1—Pd1—Cl2	95.67 (6)	C14—C15—C10	119.9 (2)
N2—Pd1—Cl1	96.49 (6)	C14—C15—H15	120.0
N1—Pd1—Cl1	172.74 (6)	C10—C15—H15	120.0
Cl2—Pd1—Cl1	89.02 (2)	C17—C16—C21	119.8 (2)
C1—N1—C3	120.6 (2)	C17—C16—C2	119.3 (2)
C1—N1—Pd1	115.20 (17)	C21—C16—C2	120.8 (2)
C3—N1—Pd1	124.03 (15)	C18—C17—C16	119.9 (2)
C2—N2—C22	119.9 (2)	C18—C17—H17	120.1
C2—N2—Pd1	115.61 (16)	C16—C17—H17	120.1
C22—N2—Pd1	123.53 (15)	C19—C18—C17	120.2 (3)
N1—C1—C10	125.8 (2)	C19—C18—H18	119.9
N1—C1—C2	114.2 (2)	C17—C18—H18	119.9
C10—C1—C2	120.0 (2)	C20—C19—C18	119.9 (2)
N2—C2—C16	123.2 (2)	C20—C19—H19	120.1
N2—C2—C1	114.7 (2)	C18—C19—H19	120.1
C16—C2—C1	121.9 (2)	C19—C20—C21	120.5 (3)
C4—C3—C8	120.8 (2)	C19—C20—H20	119.8
C4—C3—N1	117.3 (2)	C21—C20—H20	119.8
C8—C3—N1	121.9 (2)	C20—C21—C16	119.7 (3)
C3—C4—C5	119.2 (2)	C20—C21—H21	120.1
C3—C4—H4	120.4	C16—C21—H21	120.1
C5—C4—H4	120.4	C23—C22—C27	121.0 (2)
C4—C5—C6	121.3 (2)	C23—C22—N2	120.8 (2)
C4—C5—H5	119.4	C27—C22—N2	118.1 (2)
C6—C5—H5	119.4	C22—C23—C24	119.1 (2)
C5—C6—C7	118.3 (2)	C22—C23—H23	120.5
C5—C6—C9	120.8 (2)	C24—C23—H23	120.5
C7—C6—C9	120.8 (2)	C23—C24—C25	121.1 (2)
C8—C7—C6	121.4 (2)	C23—C24—H24	119.5
C8—C7—H7	119.3	C25—C24—H24	119.5
C6—C7—H7	119.3	C26—C25—C24	118.5 (2)
C3—C8—C7	118.8 (2)	C26—C25—C28	120.3 (2)
C3—C8—H8	120.6	C24—C25—C28	121.2 (2)
C7—C8—H8	120.6	C25—C26—C27	121.5 (2)
C6—C9—H9A	109.5	C25—C26—H26	119.2
C6—C9—H9B	109.5	C27—C26—H26	119.2
H9A—C9—H9B	109.5	C26—C27—C22	118.8 (2)
C6—C9—H9C	109.5	C26—C27—H27	120.6
H9A—C9—H9C	109.5	C22—C27—H27	120.6
H9B—C9—H9C	109.5	C25—C28—H28A	109.5
C11—C10—C15	119.5 (2)	C25—C28—H28B	109.5
C11—C10—C1	121.9 (2)	H28A—C28—H28B	109.5
C15—C10—C1	118.6 (2)	C25—C28—H28C	109.5
C12—C11—C10	119.9 (2)	H28A—C28—H28C	109.5
C12—C11—H11	120.0	H28B—C28—H28C	109.5
C10—C11—H11	120.0	C29—O1—H10H	105 (2)
C13—C12—C11	120.4 (3)	O1—C29—H29A	109.5
C13—C12—H12	119.8	O1—C29—H29B	109.5
C11—C12—H12	119.8	H29A—C29—H29B	109.5
C14—C13—C12	120.0 (2)	O1—C29—H29C	109.5
C14—C13—H13	120.0	H29A—C29—H29C	109.5
C12—C13—H13	120.0	H29B—C29—H29C	109.5
C13—C14—C15	120.2 (3)		
			
N2—Pd1—N1—C1	10.15 (16)	N1—C1—C10—C15	−126.3 (3)
Cl2—Pd1—N1—C1	−173.22 (16)	C2—C1—C10—C15	56.1 (3)
N2—Pd1—N1—C3	−174.59 (18)	C15—C10—C11—C12	−0.2 (3)
Cl2—Pd1—N1—C3	2.04 (17)	C1—C10—C11—C12	179.5 (2)
N1—Pd1—N2—C2	−7.07 (17)	C10—C11—C12—C13	1.0 (4)
Cl1—Pd1—N2—C2	167.06 (16)	C11—C12—C13—C14	−0.7 (4)
N1—Pd1—N2—C22	161.36 (19)	C12—C13—C14—C15	−0.4 (4)
Cl1—Pd1—N2—C22	−24.50 (18)	C13—C14—C15—C10	1.2 (4)
C3—N1—C1—C10	−4.4 (3)	C11—C10—C15—C14	−0.9 (3)
Pd1—N1—C1—C10	171.05 (18)	C1—C10—C15—C14	179.4 (2)
C3—N1—C1—C2	173.36 (19)	N2—C2—C16—C17	56.3 (3)
Pd1—N1—C1—C2	−11.2 (2)	C1—C2—C16—C17	−128.8 (2)
C22—N2—C2—C16	9.6 (3)	N2—C2—C16—C21	−122.0 (3)
Pd1—N2—C2—C16	178.51 (18)	C1—C2—C16—C21	52.8 (3)
C22—N2—C2—C1	−165.5 (2)	C21—C16—C17—C18	1.0 (3)
Pd1—N2—C2—C1	3.3 (3)	C2—C16—C17—C18	−177.4 (2)
N1—C1—C2—N2	5.3 (3)	C16—C17—C18—C19	0.2 (4)
C10—C1—C2—N2	−176.8 (2)	C17—C18—C19—C20	−1.2 (4)
N1—C1—C2—C16	−170.0 (2)	C18—C19—C20—C21	1.0 (4)
C10—C1—C2—C16	7.9 (3)	C19—C20—C21—C16	0.2 (4)
C1—N1—C3—C4	−119.0 (2)	C17—C16—C21—C20	−1.2 (4)
Pd1—N1—C3—C4	66.0 (3)	C2—C16—C21—C20	177.2 (2)
C1—N1—C3—C8	61.1 (3)	C2—N2—C22—C23	−115.9 (3)
Pd1—N1—C3—C8	−113.9 (2)	Pd1—N2—C22—C23	76.1 (3)
C8—C3—C4—C5	−4.1 (4)	C2—N2—C22—C27	65.1 (3)
N1—C3—C4—C5	176.0 (2)	Pd1—N2—C22—C27	−102.8 (2)
C3—C4—C5—C6	1.1 (4)	C27—C22—C23—C24	−1.9 (4)
C4—C5—C6—C7	2.6 (4)	N2—C22—C23—C24	179.2 (2)
C4—C5—C6—C9	−174.5 (2)	C22—C23—C24—C25	0.2 (4)
C5—C6—C7—C8	−3.5 (4)	C23—C24—C25—C26	0.9 (4)
C9—C6—C7—C8	173.6 (2)	C23—C24—C25—C28	−178.6 (2)
C4—C3—C8—C7	3.3 (4)	C24—C25—C26—C27	−0.3 (4)
N1—C3—C8—C7	−176.8 (2)	C28—C25—C26—C27	179.2 (2)
C6—C7—C8—C3	0.6 (4)	C25—C26—C27—C22	−1.3 (4)
N1—C1—C10—C11	54.0 (3)	C23—C22—C27—C26	2.4 (4)
C2—C1—C10—C11	−123.6 (3)	N2—C22—C27—C26	−178.6 (2)

### Hydrogen-bond geometry (Å, º)

**Table:**

*D*—H···*A*	*D*—H	H···*A*	*D*···*A*	*D*—H···*A*
O1—H10*H*···Cl2	0.83 (2)	2.36 (2)	3.161 (2)	163 (3)
C17—H17···Cl2^i^	0.95	2.80	3.708 (3)	161
C21—H21···O1^ii^	0.95	2.48	3.275 (3)	141

Symmetry codes: (i) −*x*+1, −*y*+1, −*z*+1; (ii) −*x*+2, −*y*+1, −*z*+1.
