# Supplementary material for: Crystal structure of [N,N′-bis­(4-methyl­phen­yl)-1,2-di­phenyl­ethane-1,2-di­imine-κ2 N,N′]di­chlorido­palladium(II) methanol monosolvate
Source: Acta Crystallogr E Crystallogr Commun. 2015 Aug 15;71(Pt 9):m164–5. doi: 10.1107/S2056989015014851 (PMC4555432; doi:10.1107/S2056989015014851)
Supplement: Supplementary file 3 [file e-71-0m164-sup3.pdf]

S1. Experimental

Synthetic procedures were carried out using standard techniques. Solvents and reagents were purchased from Sigma-Aldrich or Acros Organics and used as received. The IR spectrum was recorded from a KCl disk using a Perkin-Elmer Spectrum 2 spectrometer. The electronic absorption spectrum was recorded at room temperature from a 1×10^{-5} M chloroform solution of the title compound on a Hewlett Packard 8453 diode array spectrometer.

0.086 g (0.257 mmol, 1 eq) of $PdCl_2(DMSO)_2$ and 0.100 g (0.257 mmol, 1 eq) of *N*,*N′*-di(4-methylphenyl)-1,2-diphenylethane-1,2-diimine were combined with 10 mL of methanol and stirred for 1.5 hours at room temperature. The orange precipitate that formed was collected via vacuum filtration, washed well with water and then air-dried giving 0.0363 g (25%) of the title compound. Slow evaporation of the reaction mixture gave x-ray quality crystals of the title compound. MP: >250 °C. IR (KCl): 3135, 2922, 1514 cm^{-1}. UV-Vis (λ nm (ε)): 242 (41,200), 264 (34,800), 317 (17,800). TLC (alumina, ethanol): $R_f = 0.59$.

S2. Comment

Palladium(II) complexes prepared from α-diimine ligands have been extensively used as catalysts for the polymerization of α-olefins (Johnson *et al*., 1995; Popeney *et al*., 2005).

The asymmetric unit of the title compound consisted of one molecule of the metal complex and one molecule of methanol. The diimine core of the palladium(II) metal complex deviated slightly from planarity, with an N1–C1–C2–N2 torsion angle of 5.3 (3) °. Delocalization in the diimine core of the title compound was indicated by N–C bonds (N1–C1 at 1.299 (3) Å and N2–C2 at 1.300 (3) Å) and a C–C (C1–C2 at 1.489 (3) Å) bond that were

respectively longer and shorter than those observed in related uncomplexed diimines (Zhao *et al.*, 2015; Wang *et al.*, 2012). The delocalization observed in the diimine core of the title compound is similar to that observed in related compounds (Kubota *et al.*, 2013; Comerlato *et al.*, 2001).

The non-ideal square planar geometry of the palladium(II) core was attributed to the small, 79.13 (8) ° (N2–Pd1–N1), bite angle of the diimine chelate. Other bond angles around the palladium atom were 94.49 (6) ° (N2–Pd1–Cl1), 89.02 (2) ° (Cl2–Pd1–Cl1), and 95.67 (6) ° (N1–Pd1–Cl2). Palladacycle torsion angles of −173.22 (16) ° (Cl2–Pd1–N1–C1) and 167.06 (16) ° (Cl2–Pd1–N1–C1) were further indicators of the non-ideal square planar geometry of the central palladium atom. Similar deviation from non-ideal square planar geometry has been noted in similar compounds (Kubota *et al*., 2013; Dyankonenko *et al*., 2015).

The *N*-phenyl rings, (C22–C27) and (C3-C8), of the title compound were at an angles of 70.52 (2) ° and 65.71 (2) °, respectively, to the central palladacycle (Pd1–N1–C1–C2–N2).

The crystal packing of the title compound engaged in three different modes of weak, intermolecular hydrogen bonding. The first type noted was O–H•••Cl hydrogen bonding between a hydroxyl hydrogen atom (O1H) and a chlorine atom (Cl2) with a H•••Cl distance of approximately 2.4 Å, an O1–Cl2 distance of 3.161 (2) Å, and an O1–H•••Cl2 bond angle of 162.54 (2) °. The second type noted was C–H•••O hydrogen bonding between an aromatic hydrogen atom (C11H) and a methanol oxygen atom (O1) with a H•••O distance of approximately 2.7 Å, a C11–O1 distance of 3.202 (2) Å, and an C11–H•••O1 bond angle of 116.33 (2) °. The third type noted was C–H•••Cl hydrogen bonding between an aromatic hydrogen atom (C17H) and a chlorine atom (Cl2) with a H•••Cl distance of approximately 2.8 Å, an C17–Cl2 distance of 3.708 (2) Å, and an C17–H•••Cl2 bond angle of 161.33 (2) °. These

intermolecular interactions result in the formation of a dimer of the title compound with an inversion center (½, ½, ½).
