# Supplementary figures and images for: Crystal structure of [N,N′-bis­(4-methyl­phen­yl)-1,2-di­phenyl­ethane-1,2-di­imine-κ2 N,N′]di­chlorido­palladium(II) methanol monosolvate
Source: Acta Crystallogr E Crystallogr Commun. 2015 Aug 15;71(Pt 9):m164–5. doi: 10.1107/S2056989015014851 (PMC4555432; doi:10.1107/S2056989015014851)

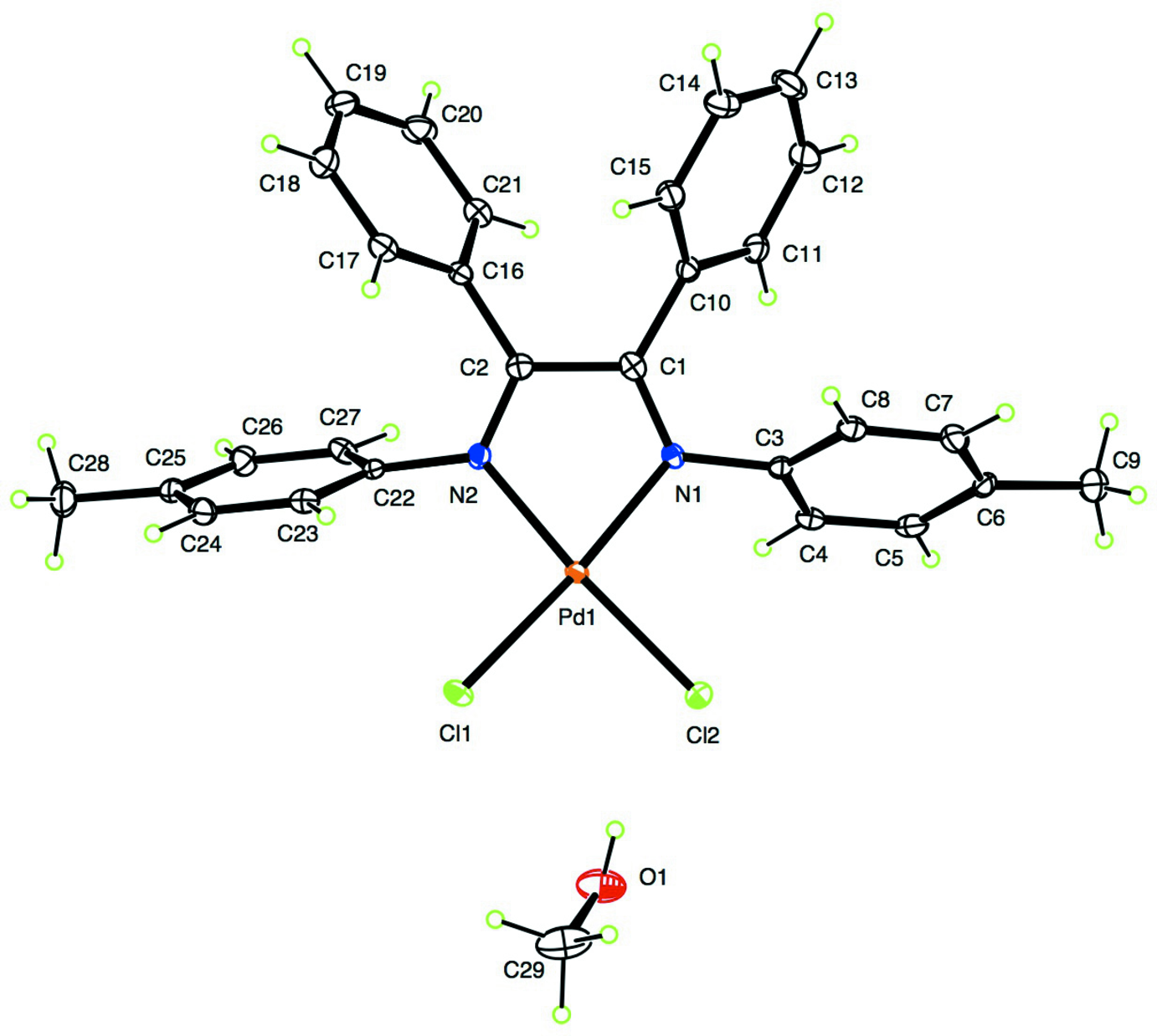

Supplement: Supplementary file 4 [file e-71-0m164-fig1.tif]

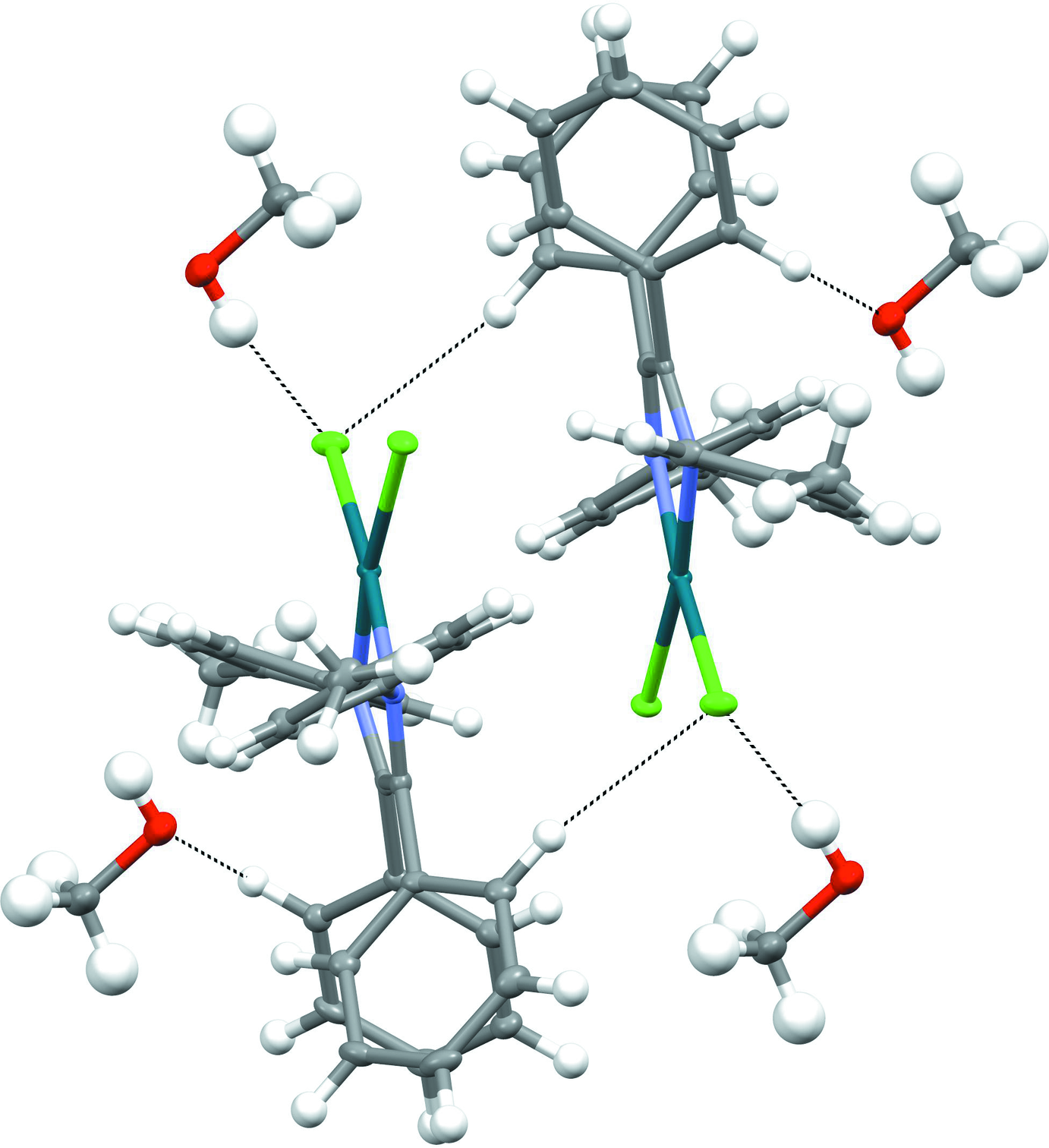

Supplement: Supplementary file 5 [file e-71-0m164-fig2.tif]

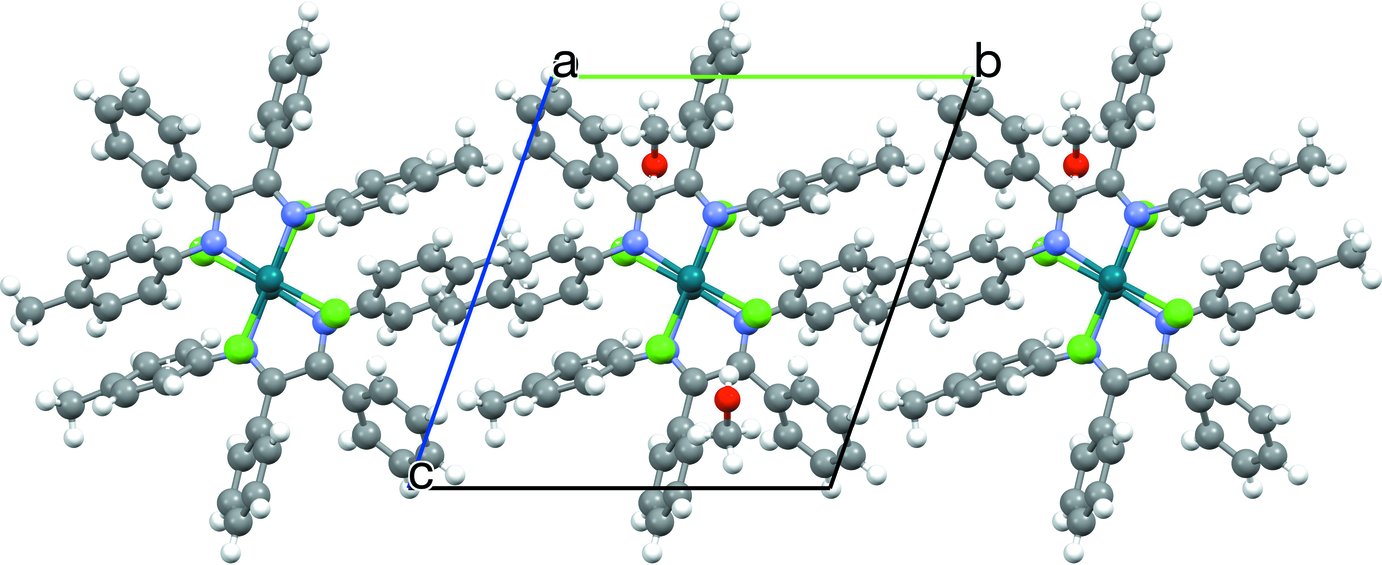

Supplement: Supplementary file 6 [file e-71-0m164-fig3.tif]
